# Comprehensive screening strategy coupled with structure-guided engineering of l-threonine aldolase from *Pseudomonas putida* for enhanced catalytic efficiency towards l-*threo*-4-methylsulfonylphenylserine

**DOI:** 10.3389/fbioe.2023.1117890

**Published:** 2023-01-30

**Authors:** Lihong Li, Rongzhen Zhang, Yan Xu, Wenchi Zhang

**Affiliations:** ^1^ Lab of Brewing Microbiology and Applied Enzymology, School of Biotechnology and Key Laboratory of Industrial Biotechnology of Ministry of Education, Jiangnan University, Wuxi, China; ^2^ Solomon H. Snyder Department of Neuroscience, Johns Hopkins University School of Medicine, Baltimore, MD, United States

**Keywords:** threonine aldolases, *Pseudomonas putida*, high-throughput screening, structure-guided engineering, product enantioselectivity

## Abstract

l-Threonine aldolases (TAs) can catalyze aldol condensation reactions to form β-hydroxy-α-amino acids, but afford unsatisfactory conversion and poor stereoselectivity at the C_β_ position. In this study, a directed evolution coupling high-throughput screening method was developed to screen more efficient l-TA mutants based on their aldol condensation activity. A mutant library with over 4000 l-TA mutants from *Pseudomonas putida* were obtained by random mutagenesis. About 10% of mutants retained activity toward 4-methylsulfonylbenzaldehyde, with five site mutations (A9L, Y13K, H133N, E147D, and Y312E) showing higher activity. Iterative combinatorial mutant A9V/Y13K/Y312R catalyzed l-*threo*-4-methylsulfonylphenylserine with a 72% conversion and 86% diastereoselectivity, representing 2.3-fold and 5.1-fold improvements relative to the wild-type. Molecular dynamics simulations illustrated that additional hydrogen bonds, water bridge force, hydrophobic interactions, and π-cation interactions were present in the A9V/Y13K/Y312R mutant compared with the wild-type to reshape the substrate-binding pocket, resulting in a higher conversion and C_β_ stereoselectivity. This study provides a useful strategy for engineering TAs to resolve the low C_β_ stereoselectivity problem and contributes to the industrial application of TAs.

## Introduction

Pyridoxal-5-phosphate (PLP)-dependent threonine aldolase (TA, EC 4.1.2.5) catalyzes the reversible aldol condensation reaction of glycine and aldehydes to form β-hydroxy-α-amino acids in a single step (Contestabile et al., 2001; Lypetska et al., 2014). β-Hydroxy-α-amino acids are important chiral building blocks for the preparation of agrochemicals and pharmaceuticals bioactive products (Steinreiber et al., 2007; Hibi et al., 2015). For example, l-*threo*-4-methylsulfonylphenylserine (MSPS) is a key precursor for the production of antibiotics, thiamphenicol and florfenicol (Zhao et al., 2011), and l-*threo*-3,4-dihydroxyphenylserine is an anti-Parkinsonism drug approved by Food and Drug Administration (Zhao et al., 2021).

**FIGURE 1:**
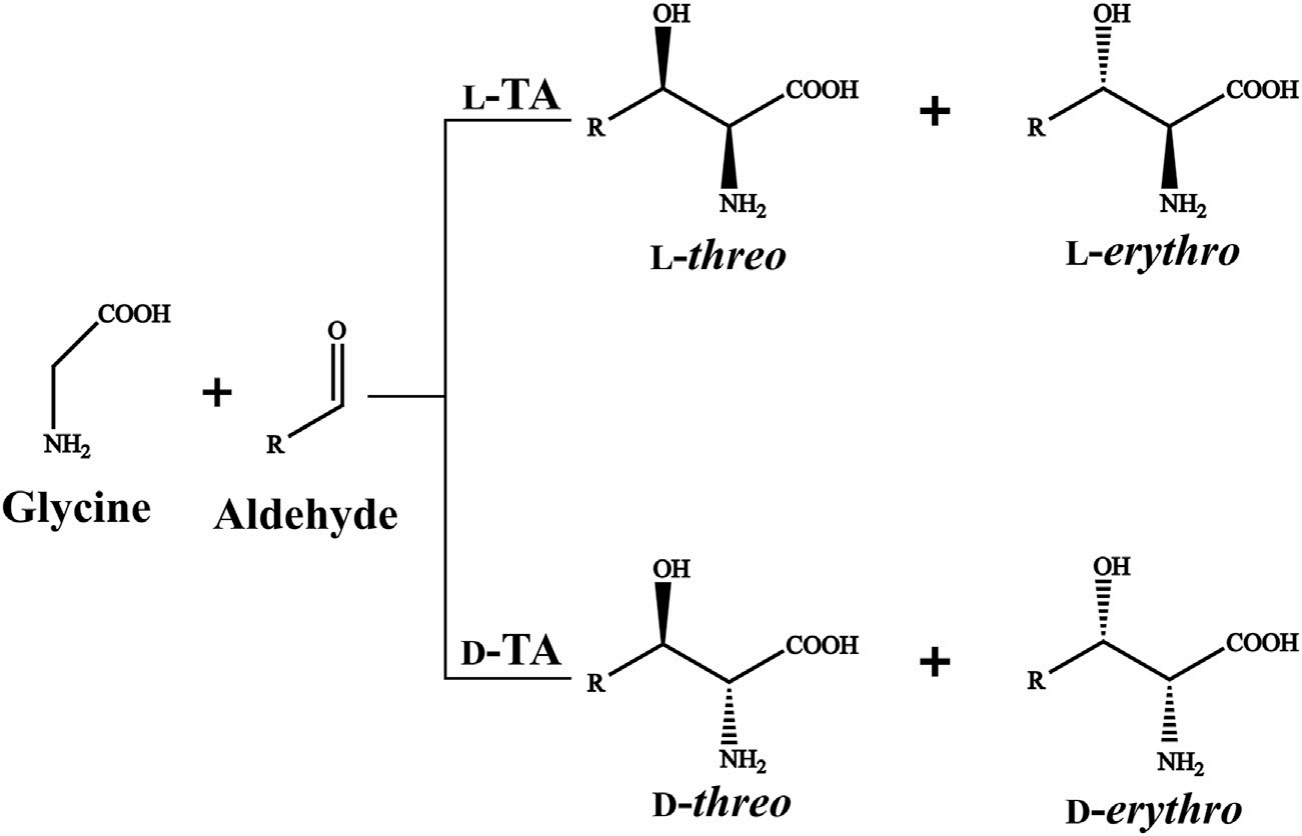
l-TA/d-TA catalyzed reaction.

TA-mediated β-hydroxy-α-amino acids biosynthesis is regarded as a feasible alternative owing to their high stereoselectivity, uncomplicated reaction procedure and environmental friendliness. TAs consist of l-TA and d-TA based on the C_α_ stereospecificity of the products (Fesko et al., 2015). l-TAs generate the l-*threo* and l-*erythro* products, while d-TAs form d-*threo* and d-*erythro* products (Figure 1). Most of TAs have an excellent stereoselectivity towards C_α_ but exhibit poor stereoselectivity at the C_β_ (Duckers et al., 2010; Wang et al., 2021). The inadequate C_β_ stereoselectivity has limited the application of TAs in industrial production (Fesko et al., 2018). Thus, it is urgent to develop the novel TAs with good stereoselectivity at the C_α_ and C_β_ simultaneously using a rapid and reliable method.

Recently, substantial attempts have been devoted for engineering TAs to enhance the stereoselectivity at the C_β_. Fesko et al. (2008) labeled 4 TAs with ^13^C- distribution in the retro-aldol reaction and determined the formation of glycine–PLP quinonoid complex. (Liu Z. C. et al., 2020) found that residues external the catalytic pocket also affected the C_β_ stereoselectivity of TA from *Pseudomonas* sp. And Chen et al. (2019) developed a substrate-binding-guided mutagenesis and stepwise visual screening method to improve or invert C_β_-stereoselectivity of TA from *Pseudomonas* sp. All reported methods represent the methodology to improve C_β_ stereoselectivity, while fundamentally resolve the “C_β_” problem requires more efforts.

Directed evolution is considered as a common means to modify the enzymes performance (Ali et al., 2020; Qu et al., 2020), such as expanding substrate spectrum (Zheng et al., 2017), enhancing catalytic functions (Wu et al., 2020), and improving stereoselectivity (Li et al., 2021). Site-directed saturation mutagenesis (SM) strategies are commonly used to mutate residues lining or adjacent the catalytic pocket, such as structure-based combinatorial protein engineering (Wang et al., 2021), combinatorial active site saturation test (Reetz et al., 2005) and iterative combinatorial mutagenesis (ICM) (Liu Y. F. et al., 2020). The directed evolution and site-mutagenesis ideally in combination might be a useful strategy for improving TAs-catalyzed reaction efficiency.

Moreover, traditional enzyme activity of TAs was determined according to the retro-aldol reaction (Steinreiber et al., 2007; Fesko, 2016). In this assay, threonine is transformed into glycine and acetaldehyde by TAs. Then, yeast alcohol dehydrogenase reduces acetaldehyde to ethanol and simultaneously oxidizes NADH to NAD^+^, monitoring the decrease of absorbance at 340 nm. However, in this method, retro-aldol reaction replaced aldol reaction for β-hydroxy-α-amino acids synthesis, and threonine as model substrate substituted the actually used substrate. In view of the application, the properties of variants on the product synthesis are far more relevant (Giger et al., 2012; Liu et al., 2014). Thus, TA evolution coupling with a high-throughput screening (HTS) method is required to be developed, which can monitor synthesis efficiency of β-hydroxy-α-amino acids by aldol reaction.

Previously, we identified an aldolase l-PpTA from *Pseudomonas putida* KT2440, which could synthesize l-*threo*-4-methylsulfonylphenylserine with glycine and 4-methylsulfonylbenzaldehyde as substrates (Li et al., 2019). In this work, we evolved l-PpTA to construct a high-quality library, and developed an efficient and sensitive HTS method basing visual color reaction of 2,4-dinitrophenylhydrazine (DNPH). The mutants with improved l-*threo*-4-methylsulfonylphenylserine biotransformation were screened basing the enzyme activity in aldol reaction direction. Then, we employed SM/ICM strategy to combine beneficial site mutations to obtain the best variant. This work will supply good protein engineering strategy for improving C_β_-stereoselectivity of TA enzymes.

## Experimental sections

### Microorganisms and chemicals

*Escherichia coli* BL21 (DE3) stored in our laboratory served as host strain. NADH, PLP and acetonitrile were purchased from Sigma-Aldrich (Shanghai, China). The PrimeSTAR DNA polymerase and restriction enzymes were purchased from Takara (Dalian, China). The ClonExpress MultiS One Step Cloning Kit was purchased from Vazyme (Nanjing, China). l-*threo*-4-methylsulfonylphenylserine was purchased from Baishansheng Bio-Technology Co., Ltd (Hangzhou, China). All other chemicals used were of the highest grade and commercially available.

### Construction of recombinant strain with _L_-PpTA

The gene *latE* (GenBank accession no. AE015451.2) coding for l-TA from *Pseudomonas putida* KT2440 (l-PpTA) was chemically synthesized by Shenggong Co. Ltd. (Shanghai, China). It was cloned into the vector pET28a in *E. coli* BL21 (DE3). Recombinant *E. coli* BL21/pET28a-l-PpTA was acquired after DNA sequencing.

### Construct mutant library by error prone PCR

Error-prone PCR of l-PpTA was carried out using the recombinant plasmid pET28a-l-PpTA as a template. The reaction mixture (50 μl) contains reaction buffer, 2.5 mM dNTP mixture, 100 μM MnSO_4_, 500 μM MgCl_2_, 0.1 μM per primer pair, 10 ng template and 1.25 U rTaq polymerase and ultra-pure water. Forward primers were 5′- AGC​AAA​TGG​GTC​GCG​GAT​CCA​TGA​CCG​ATA​AAA​GCC​AGC​AG-3′ and reverse primers were 5′-GGT​GGT​GGT​GGT​GCT​CGA​GTT​AGC​CAC​CGA​TGA​TG GTACG-3′. PCR was performed at 98 °C for 30 s, 55°C for 30 s, and 72°C for 60 s for 34 cycles. The PCR products were cloned into the pET28a vector between *Bam*H I and *Xho* I sites using Exnase ClonExpress MultiS One Step Cloning Kit, followed by transformation into the competent cells of *E. coli* BL21 (DE3). The recombinant *E. coli* cells were cultured on LB plates added with 50 μg⋅ml^−1^ kanamycin and incubated overnight at 37°C to obtain the mutant library.

The colonies on LB plates were displaced into 96-well plates (each containing 500 μl LB medium and 50 μg⋅ml^−1^ kanamycin), and incubated at 37°C for 3 h. 0.1 mM IPTG was added to induce the protein expression at 25°C for 14 h. The cells were collected by centrifugation with 6,000 × g for 5 min.

### HTS of the mutant library by DNPH screening method

The enzyme assay mixture (200 μl) contained 100 mM HEPES-NaOH buffer (pH 8.0), appropriate amount of enzyme solution, 150 μl of 2.5 mM 4-methylsulfonylbenzaldehyde (MSBA) dissolved with 10% DMSO, 30 μl of 25 mM glycine and 10 μl of 50 μM PLP. The reaction mixture was shaken at 200 rpm, 30°C for 5 min. Color reaction was carried out as follows: 10 μl of the above-mentioned reaction mixture was added into 90 μl of 20 mM 2,4-dinitrophenylhydrazine (DNPH) dissolved in 10% HCl, and the reaction was performed for 1 min to obtain the white sediment. And 100 μl of 4 M NaOH was added into the white sediment, resulting in the red-brown solution. The absorbance of the red-brown solution was determined at 470 nm by spectrophotometer (Biotek, Vermont, United States). To achieve high detection sensitivity, the effect of incubation time (1, 2, 3, 4, 5, 10, 20 and 30 min) and alkalis (NaOH, KOH, Na_2_CO_3_, K_2_CO_3_, Na_2_SO_4_, K_2_SO_4_, NaHCO_3_, and KHCO_3_) with different pK_b_ values were determined.

The standard curve of MSBA using DNPH screening method was made with different concentrations of MSBA (0.25–5.0 mM) dissolved with 10% DMSO in 100 mM HEPES-NaOH buffer (pH 8.0) at 30°C and with a shaken at 200 rpm for 5 min. Then, the absorbance is determined by color reaction method.

The collected cells were suspended in 1 ml of HEPES-NaOH buffer (100 mM, pH 8.0) and screened using DNPH method. The absorbance of the red-brown solution was determined at 470 nm by a spectrophotometer. One unit of the aldol activity (U) was defined as the amount of enzyme needed to consume 1 μmol MSBA per minute. *E. coli* BL21/pET28a was regarded as a control.

### Saturation mutations and combinatorial mutations

Five amino acid residues, A9, Y13, H133, E147, and Y312 lining the substrate-binding pocket were selected. The SM libraries of l-PpTA were constructed to encode 20 canonical amino acids using the degenerate codons NNK. The short fragments include the target mutations were amplified using the primers S-F and S-R. The long fragments were amplified using the primers L-F and primers L-R. Then, the short fragments and long fragments were connected using the ClonExpress MultiS One Step Cloning Kit through homologous recombination (Supplementary Figure S1). The primers used are listed in Supplementary Table S1. The beneficial mutants were screened using the DNPH screening methods from saturation mutation library. Then the double site combined mutants (A9V/Y13K, A9V/H133N, A9V/E147D, A9V/Y312R, Y13K/H133N, Y13K/E147D, Y13K/Y312R, H133N/E147D, H133N/Y312R, and E147D/Y312R) and the triple site combined mutants (A9V/Y13K/H133N, A9V/Y13K/E147D, A9V/Y13K/Y312R, Y13K/H133N/E147D, Y13K/E147D/Y312R, and H133N/E147D/Y312R) were also constructed using the homologous recombination approach.

### Protein expression and purification

The recombinant *E. coli* was cultured in LB medium added with 50 μg⋅ml^-1^ kanamycin at 37°C and shaken at 200 rpm. When the OD_600_ value of the culture reached 0.6–0.8, 0.1 mM IPTG was supplemented to induce protein expression at 25°C for 14 h. The cells were harvested by centrifugation and suspended in buffer (20 mM Tris–HCl and 150 mM NaCl, pH 8.0) and lysed by an ultrasonic oscillator (Sonic Materials Co., Piscataway, USA). The supernatant was gathered by centrifugation (12,000 × g, 30 min) at 4°C.

The collected supernatant was loaded on a HisTrap affinity column equilibrated with buffer (20 mM Tris–HCl and 150 mM NaCl, pH 8.0), and then it was eluted with buffer (20 mM Tris–HCl, 150 mM NaCl, 1 M imidazole, pH 8.0). Afterwards the pooled fractions were applied to a Resource Q column equilibrated with buffer (20 mM Tris–HCl, pH 8.0). Finally, the fractions were loaded on a Superdex 200 (10/300 GL) gel column for chromatography. The purified l-PpTA was assayed by sodium dodecyl sulfate-polyacrylamide gel electrophoresis (SDS-PAGE).

### Enzyme assay and kinetic determination

The enzyme activities in aldol reaction were measured on the basis of DNPH screening method. The kinetic parameters were measured at different glycine concentrations (2.5–50 mM). The *K*_M_ and *k*_cat_ values were determined by fitting the data to Michaelis−Menten equation using GraphPad Prism for non-linear regression. Each measurement was repeated for three times.

### Biotransformation and analytical methods

The asymmetric reaction was performed in 2 mL volume consisted of 50 mM potassium dihydrogen phosphate buffer (pH 8.0), 100 mM MSBA, 1 M glycine, 50 μM PLP, 10% DMSO and an appropriate amount of enzyme solution. The reactions were performed at 30°C for 8 h with shaking at 200 rpm. At the end of reaction, the reaction solution was boiled for 10 min, and then it was diluted with 50 mM potassium dihydrogen phosphate buffer (pH 8.0). The conversion and stereospecificity of product were determined by High Performance Liquid Chromatography after its derivatization with ortho-phthaldialdehyde/N-acetyl cysteine (OPA/NAC) (Steinreiber et al., 2007) on an achiral RP18 column (250 mm/5 μm). The OPA/NAC derivative reagent was acquired by dissolving 20 mg NAC in 4 mL buffer (0.2 M boric acid, pH 10.2) and then 20 mg OPA in 1 mL acetonitrile was added. The chromatographic analysis was carried out using Agilent 1260 high performance liquid chromatography system (Agilent Technologies Inc., Palo Alto, USA) with an UV detector at 338 nm. The detection conditions were performed in the mobile phase: 50 mM KH_2_PO_4_, pH 8.0/acetonitrile = (81/19); flow rate: 0.8 mL⋅min^-1^; temperature: 40°C; run time: 30 min.

### Molecular docking and molecular dynamic simulations

*P. putida* l-TA (PDB ID: 5VYE) shared the similarity of 98.26% with l-PpTA in amino acid sequence. Molecular docking of l-PpTA and its variants were built with Discovery Studio using crystal structure of *P. putida* l-TA (PDB ID: 5VYE) as template. The ligands were optimized and docked into l-PpTA and its variants by using flexible docking. Molecular dynamic (MD) simulations were carried out as follows: firstly, the CHARMm force field was applied to the protein, and the system was constructed in a cubic box composed of TIP3P water molecules that stretched 10 Å away from the protein surface to create a buffer zone between them; Secondly, sodium ions as counterions were added to the system to build a neutral system; Thirdly, the step of energy minimization was carried out using the conjugate gradient algorithms; Finally, the protein-ligand complex simulations with 50 ns were carried out, gradually heat the system from 0 to 300 K.

## Results and discussion

### Establishment of HTS method based on aldol reaction activity of l-PpTA

**FIGURE 2:**
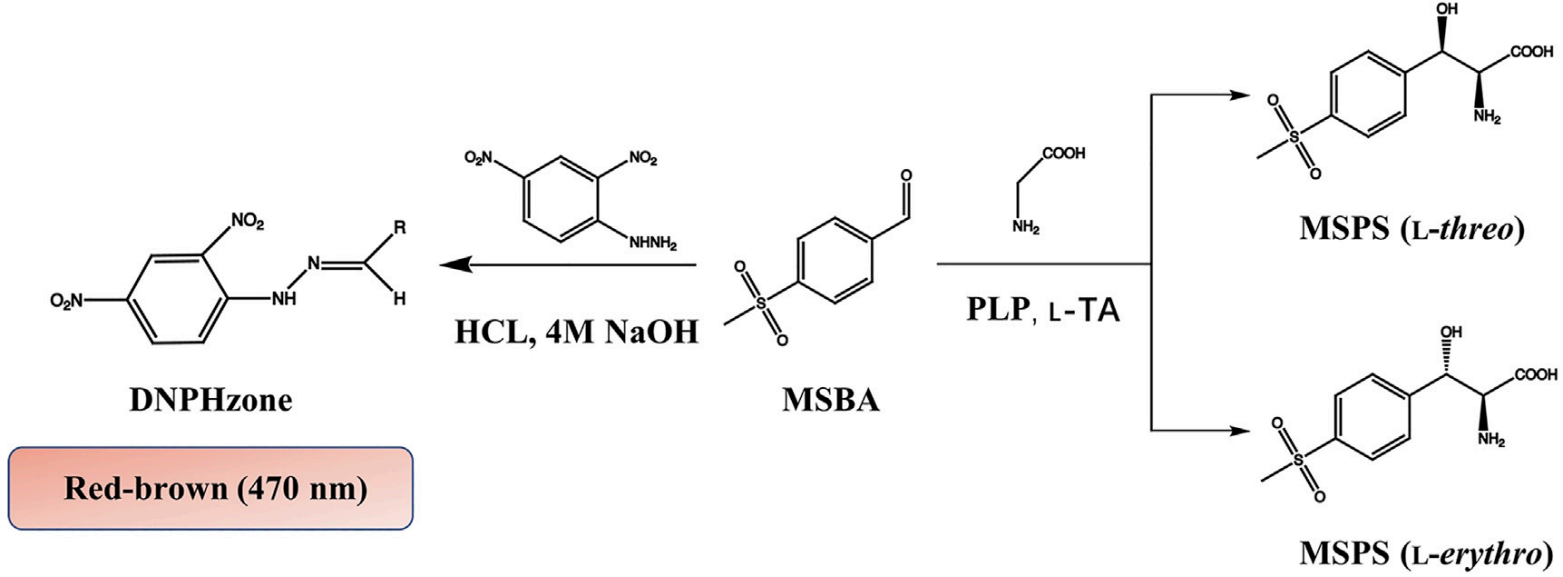
HTS colorimetric method for determining MSBA comsuption by l-PpTA.

**FIGURE 3:**
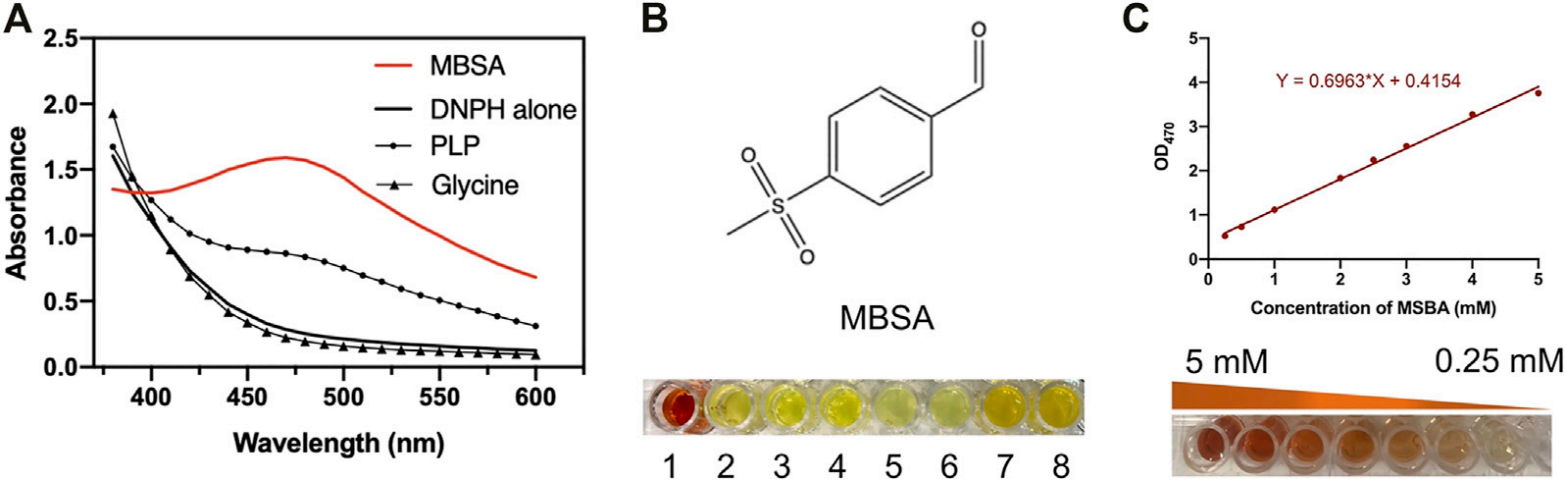
**(A)** Absorbance spectra of MSBA, PLP, Glycine, and DNPH; **(B)** Red-brown color formation between different alkalis including NaOH, KOH, Na_2_CO_3_, K_2_CO_3_, Na_2_SO_4_, K_2_SO_4_, NaHCO_3_, and KHCO_3_; **(C)** The standard curve of MSBA determined DNPH methods at OD_470_.

Recombinant *E. coli* BL21/pET28a-l-PpTA was constructed and the recombinant l-PpTA enzyme was purified. This enzyme catalyzed the transformation of substrates glycine and MSBA into MSPS owing to its aldol condensation reaction activity. MSBA is a non-natural aromatic aldehyde, whose aldehyde group can react with DNPH-zine under basic conditions to produce red-brown product DNPH-zone (Figure 2). In the l-PpTA-catalyzed aldol reaction, four potential components, namely, MSBA, DNPH, PLP, and glycine, possibly affected the absorbance. Substrate MSBA exhibited an absorbance peak at 470 nm, which was distinct from those of DNPH, PLP, and glycine (Figure 3A), indicating that the DNPH screening method was feasible for MSBA determination.

The effects of incubation time and base type on the colorimetric reaction between DNPH-zine and MSBA were investigated. Incubation for 1 min at 30°C was sufficient to produce DNPH-zone with a distinct red-brown color. Several bases with different pK_b_ values, including NaOH, KOH, Na_2_CO_3_, K_2_CO_3_, Na_2_SO_4_, K_2_SO_4_, NaHCO_3_, and KHCO_3_, were used as chromogenic reagents. The results showed that only NaOH produced the clear red-brown color (Figure 3B).

A calibration curve and detection limit were required for quantitative determination of MSBA concentration in the DNPH screening method. As shown in (Figure 3C), a linear relationship was observed in the MSBA concentration range of 0.25–5.0 mM at OD_470_, indicating the feasibility of the DNPH screening method for low-concentration MSBA. The color became light red-brown with an increase in MSBA concentration (Figure 3C). These results demonstrated that the DNPH screening method was sufficiently sensitive for the quantification of MSBA in l-PpTA screening.

### Screening five potential sites with higher aldol condensation activity using directed-evolution coupling HTS method

**FIGURE 4:**
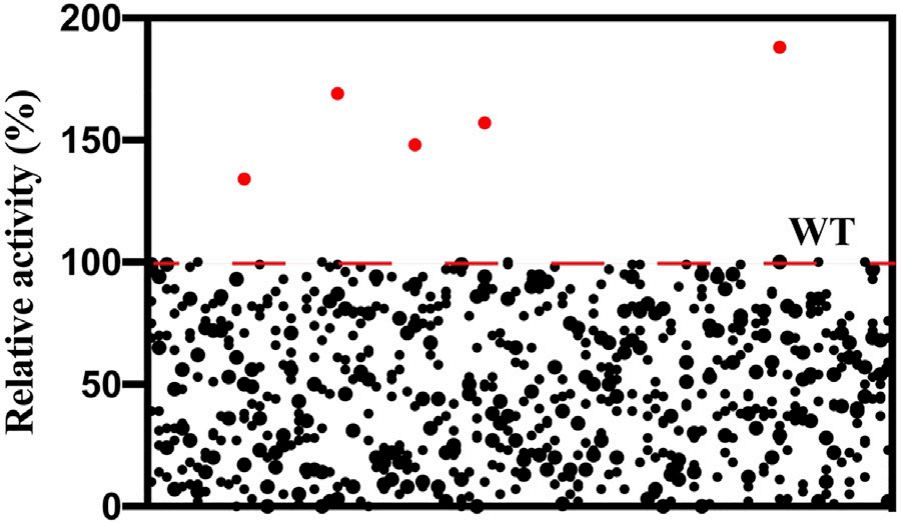
Screening of the random mutagenesis library of l-PpTA. Red dots represent the selected mutants.

To obtain variants with improved aldol activity, a random mutagenesis library of l-PpTA containing 4280 variants was constructed by the error-prone PCR technique. Selection of the beneficial mutations was attempted using the DNPH screening method (Supplementary Figure S2). The supernatant of most mutants showed reduced activity, with some completely losing activity. About 10% of the mutants retained aldol reaction activity with glycine and MSBA as substrates (Figure 4). Only five variants showed higher aldol reaction activity. After DNA sequencing, mutations A9L, Y13K, H133N, E147D, and Y312E were identified, and all were expressed as soluble forms in the recombinant *E. coli* (SupplementaryFigure S3).

**FIGURE 5:**
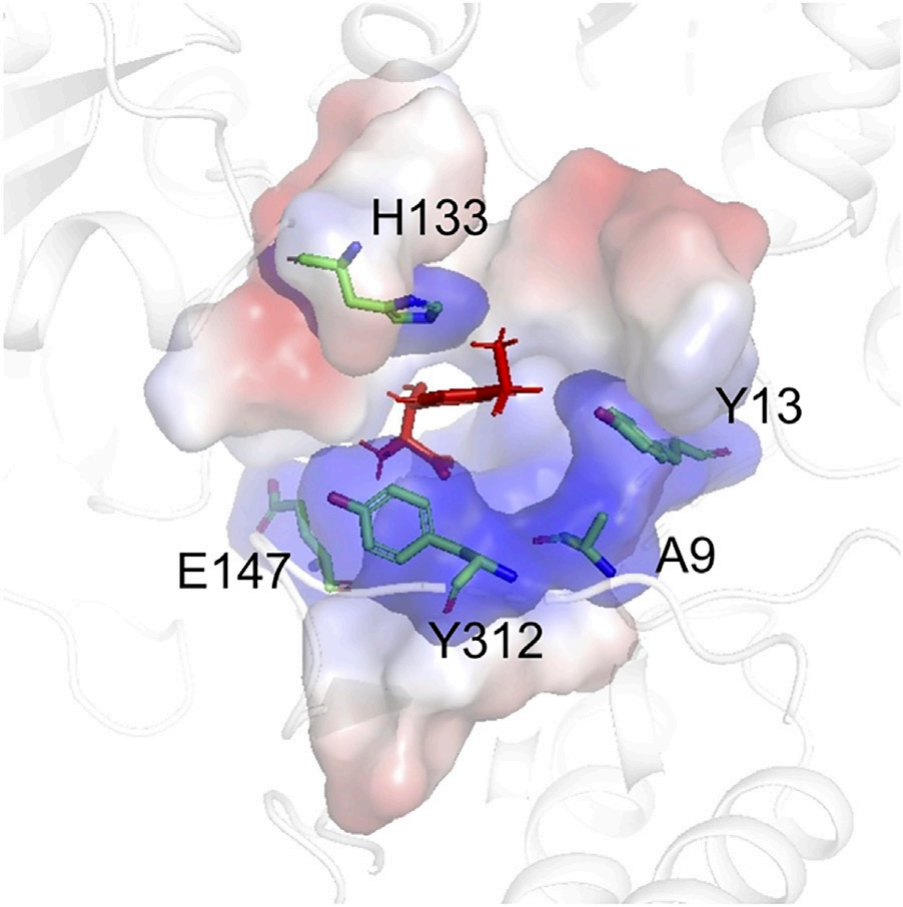
The model structure of WT l-PpTA. The enzyme backbone is represented as a cartoon in white. The substrate binding pocket is represented as a surface. The MSPS is indicated by red sticks.

**FIGURE 6:**
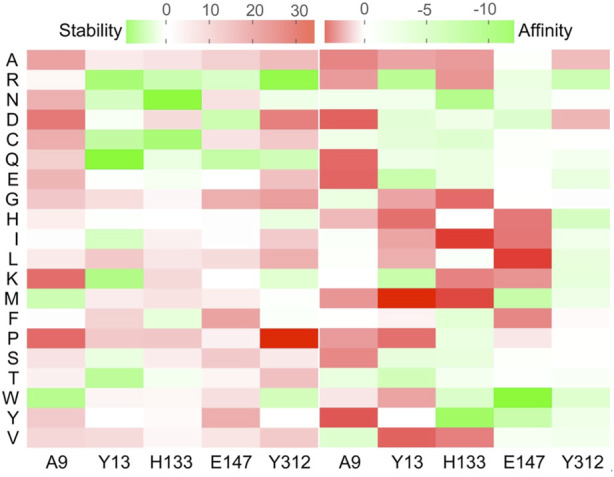
Virtual mutation of A9, Y13, H133, E147, and Y312 residues in l-PpTA.

**FIGURE 7:**
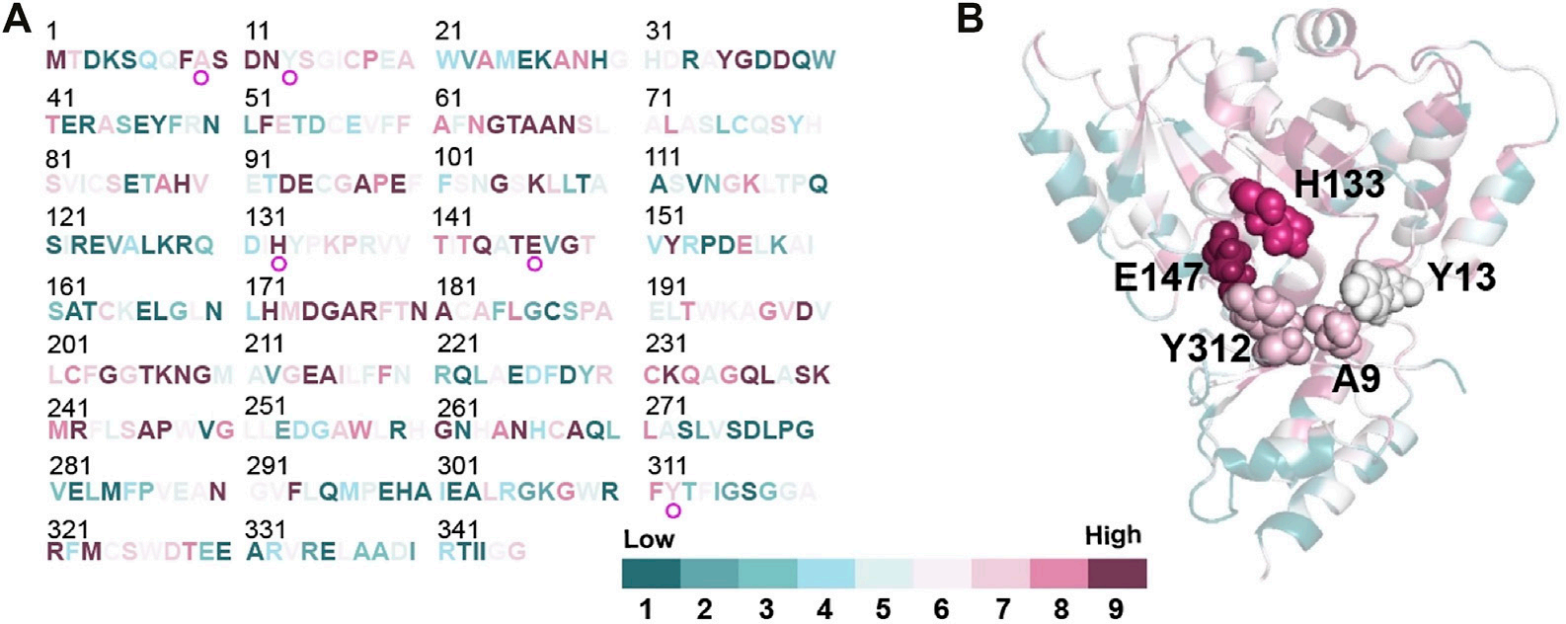
Conservation analysis of l-PpTA by ConSurf (https://consurf.tau.ac.il/). Amino acids are colored by their conservation grades. **(A)** Conservation of each amino acids residue in the protein sequence; **(B)** The protein structure of the monomer represented as a cartoon. A9, Y13, H133, E147, and Y312 are highlighted by spheres.

The structure model of l-PpTA was built based on the known crystal structure (PDB: 5VYE) and docked with product MSPS using Discovery Studio (DS) 4.1. In the modelled l-PpTA structure, residues A9, Y13, H133, E147, and Y312 were located in the catalytic pocket area (Figure 5). The stability and affinity of protein–product complexes were calculated using the DS binding mutation energy after the five amino acids were mutated to 19 other amino acid residues (Petukh et al., 2016; Zheng et al., 2020). Lower values indicated variants with more stable protein–product complexes and higher affinities. The DS algorithm results indicated that Y13 and Y312 exhibited higher stability and affinity (Figure 6). Sequence conservation analysis showed that the five residues had moderate to high conservation. H133 and E147 were the most highly conserved (Figure 7), which might provide high evolutionary selective pressure and significantly impact protein function (Chen et al., 2019). Lee et al. found that mutations at conserved positions during evolution had a greater effect on enzyme functions than those at non-conserved positions (Lee and Goodey, 2011).

### Identified beneficial mutants from saturation mutation library of five potential sites

To further improve the catalytic efficiency, an SM library of the five potential sites, A9, Y13, H133, E147, and Y312, was constructed. Thirteen positive variants, A9L, A9V, Y13C, Y13K, Y13Q, Y13R, H133N, H133Y, H133W, E147D, Y312R, Y312E, and Y312K, were obtained (Supplementary Figure S4). All variants showed a certain degree of improvement of *k*_cat_/*K*_M_ values in the aldol reaction direction. The A9V, Y13K, H133N, E147D, and Y312R mutants showed higher *k*_cat_/*K*_M_ values compared with the WT. Among all mutations, H133N resulted in the highest *k*_cat_/*K*_M_ value (2.4-fold higher than that of the WT (Table 1), suggesting that H133N had a favorable effect on the aldol reaction. Chen et al. (2019) suspected the H133 participated in regulating the C_β_ stereoselectivity by adjusting protonation of the aldehyde group in the aldol reaction. Salvo et al. (2014) found that mutations of H126 in *E. coli*_L_-TA (corresponding to H133 in l-PpTA) had an effect on establishing or destroying the hydrogen bond between H126 and the substrate hydroxyl group. Fesko mutated H128Y in *Aeromonas jandaei*_L_-TA (corresponding to H133 in l-PpTA), which improved the conversion efficiency of l-β-phenylserine in the aldol addition reaction (Fesko, 2019). As complementary experiments, H133N and H133F enhanced activity in both the retro-aldol cleavage and aldol reaction directions. Other variants clearly decreased retro-aldol cleavage activity, but improved the aldol reaction activity (data not shown). These results suggested that the traditional activity assay corresponding to retro-aldol activity could not calibrate the aldol reaction activity for the biotransformation of β-hydroxy-α-amino acids.

**TABLE 1:** Kinetic parameters of l-PpTA and its variants in aldol direction.

Enzymes	*K*_M_ (mM)	*k*_cat_ (s^-1^)	*k*_cat_/*K*_M_ (s^-1^ mM^-1^)
WT	17.4	7.1	0.41
A9L	14.6	7.7	0.53
A9V	13.8	7.2	0.52
Y13C	24.7	13.9	0.56
Y13K	19.6	10.4	0.53
Y13Q	30.8	15.4	0.50
Y13R	34.4	16.8	0.49
H133N	12.8	12.7	0.99
H133Y	22.6	12.5	0.55
H133W	12.9	12.3	0.95
E147D	15.1	6.5	0.43
Y312R	20.1	12.2	0.61
Y312E	25.3	15.3	0.60
Y312K	26.3	11.04	0.42

Using WT, A9V, Y13K, H133N, E147D, and Y312R as catalysts, the MSPS conversion and diastereomeric excess (*de*) were measured by HPLC (Table 2, Fig. S5). Y312R exhibited the highest catalytic efficiency, affording a 58% conversion and 61% *de*, which were 1.8–3.6-fold higher than those of the WT. Variants A9V and Y13K achieved a conversion of 57%–61% and 50%–51% *de* in MSPS production. H133N and E147D increased the conversion to 54% and 35%, and the *de* values to 47% and 52%, representing increases of 1.1–1.7-fold and 2.8–3.1-fold compared with the WT, respectively.

**TABLE 2:** The conversion and *de* value of positive variants produced in SM and ICM.

Enzymes	Library	*de* (%)	conv (%)
WT	—	17	32
A9V	SM	51	57
Y13K	SM	50	61
H133N	SM	47	54
E147D	SM	52	35
Y312R	SM	61	58
A9V/Y13K	ICM	65	64
A9V/Y312R	ICM	71	59
Y13K/Y312R	ICM	79	61
A9V/Y13K/Y312R	ICM	86	72

### Significantly improved catalytic efficiency of MSPS by combinatorial mutagenesis

To further enhance the catalytic efficiency of l-PpTA, the iterative combinatorial mutagenesis strategy was conducted on the five single mutants (A9V, Y13K, H133N, E147D, and Y312R). Three high-performing double-site combined mutants, A9V/Y13K, A9V/Y312R, and Y13K/Y312R with improved catalytic efficiency were identified (Table 2). Among them, variant Y13K/Y312R exhibited the highest catalytic efficiency, with a conversion of 61% and 79% *de*, representing 1.9-fold and 4.6-fold improvements, respectively. Furthermore, A9V/Y13K and A9V/Y312R catalyzed the synthesis of MSPS with conversion of 59%–64% and *de* values of 65%–71%, while the WT only afforded the product with a 32% conversion and 17% *de*. The other variants with double-site combined mutations, namely, A9V/H133N, A9V/E147D, Y13K/H133N, Y13K/E147D, H133N/E147D, H133N/Y312R, and E147D/Y312R improved the conversion 1.3–1.8-fold and the *de* value 2.6–3.5-fold (Supplementary Figure S5).

Among the triple-site combined mutants, A9V/Y13K/Y312R achieved the highest catalytic efficiency, which converted 100 mM MSBA and 1 M glycine to MSPS after reacted 30°C for 8 h in pH 8.0 with a conversion of 72% and 86% *de*, (Supplementary Figure S6), representing 2.3-fold and 5.1-fold improvements, respectively (Table 2).

The combined mutants involving E147D (A9V/Y13K/E147D, Y13K/H133N/E147D, Y13K/E147D/Y312R, and H133N/E147D/Y312R) did not lead to obviously enhanced efficiency, with conversion of 51%–58% and *de* values of 56%–71%. Furthermore, combination A9V/Y13K/H133N showed a conversion of 61% and 69% *de*, which were 1.9-fold and 4.1-fold higher than those of the WT, respectively. Among all combinatorial mutations, A9V/Y13K/Y312R resulted in the highest values of 86% *de* with a 72% yield, which were also higher than previously reported results (Liu Z. C. et al., 2020).

### Mechanism of improved catalytic efficiency from molecular dynamics simulations

**FIGURE 8:**
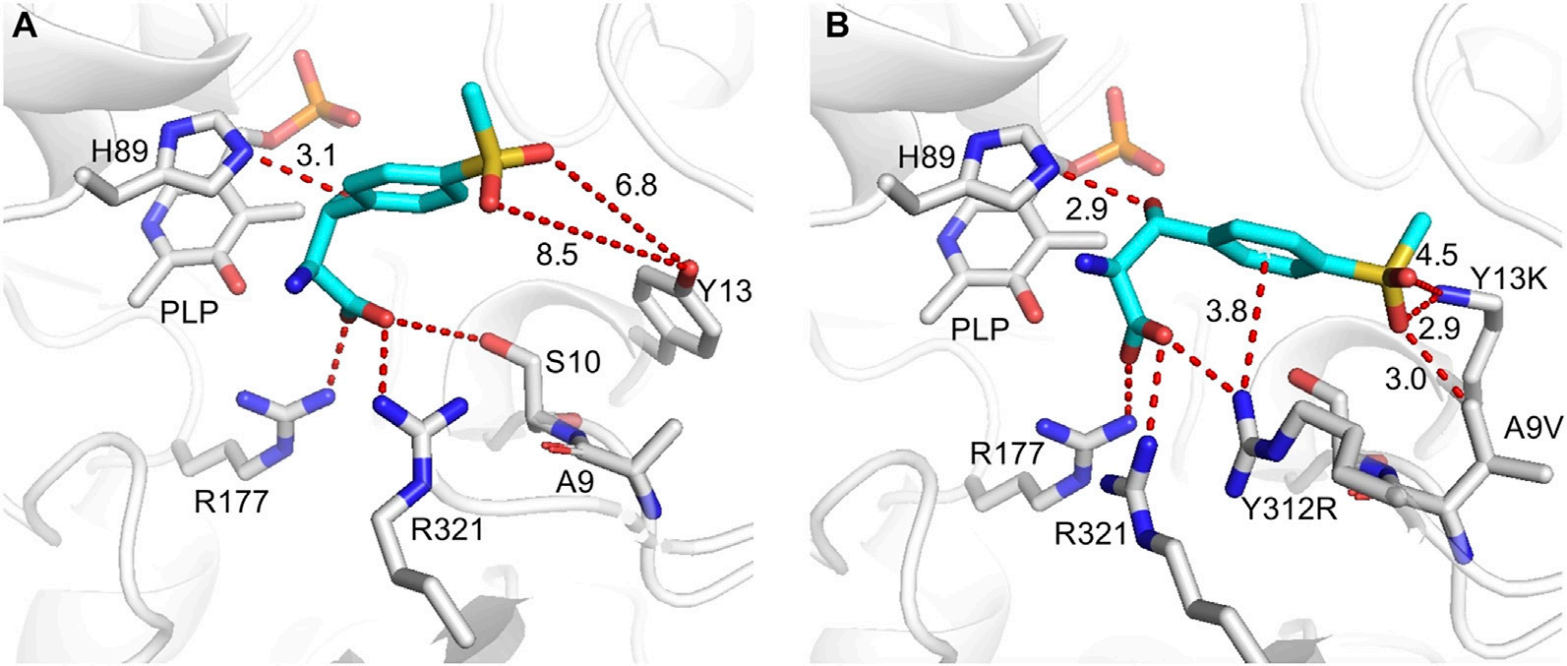
Flexible docking results of MSPS (cyan) in WT **(A)** and A9V/Y13K/Y312R **(B)**. Active sites and PLP are displayed in grey, the interactions are indicated by red dashes.

To gain molecular insight into the enhanced catalytic efficiency produced by A9V/Y13K/Y312R mutations, the l-PpTA structure was modeled using the *P. putida* l-TA complexed structure with PLP (PDB ID: 5VYE) as a template, and product MSPS was docked into the active center of the WT and its variants. In all docked structures, a conserved H89 stack was parallel to the PLP ring, forming a π–π interaction with cofactor PLP. The distance between H89-NE2 and the hydroxyl group of MSPS was 2.9–3.1 Å, which was within hydrogen-bonding distance, suggesting that H89 acted as the catalytic base to initiate the aldol reaction (Wang et al., 2021). The carboxylate group of MSPS formed a salt bridge with the side chains of highly conserved R177, R321, and S10 (Figure 8; Supplementary Figure S7) to anchor the carboxylate of MSPS and equilibrate the transient state generated during the reaction (Salvo et al., 2014). In the WT docking conformation, the benzene ring and sulfonyl group of MSPS were free, without direct and strong anchoring forces (Figure 8A), while the A9V variant formed a powerful hydrophobic interaction between the methyl group of V9 and sulfonyl group of MSPS (Supplementary Figure S8A). The Y13K mutation in the substrate binding pocket showed a shortened distance (decreased from 6.8 to 8.5 Å to 2.8–4.7 Å) between its amino group and the two oxygen atoms of MSPS-RSO_2_ (Supplementary Figure S8B). Furthermore, the Y312R mutation resulted in a newly formed π-cation interaction between the guanidine group of R312 and the benzene ring of MSPS, and a salt bridge between the guanidine group of R312 and the carboxylate group of MSPS, which enhanced their attractive charge interaction and conformation stability (Supplementary Figure S8C). In the mutant A9V/Y13K/Y312R structure model (Figure 8B), three amino acids were adjacent to the benzene ring and sulfonyl group of the ligand. Among them, K13 and R312 were polar amino acids with big side chain, which dramatically shrank the binding-pocket and enhanced the electrostatic interactions with the ligand. Simultaneously, the mutant V9 supplemented the hydrophobic interaction with the ligand. Therefore, the binding pocket was reshaped after the mutation, which would contribute to improve the catalytic efficiency.

**FIGURE 9:**
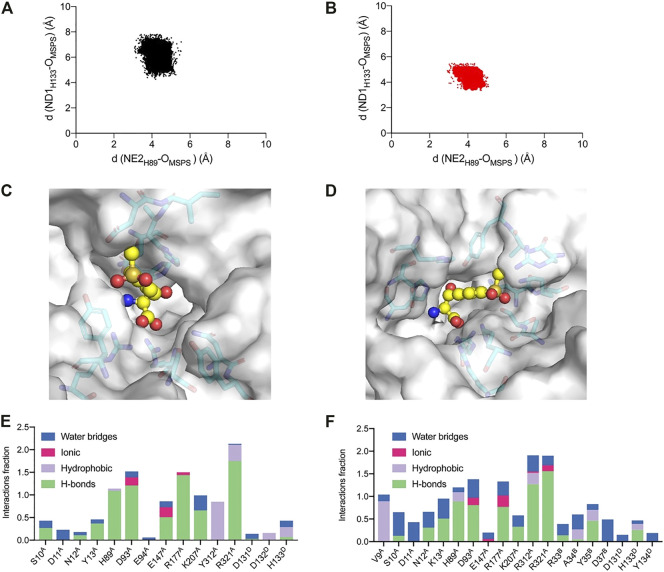
Analysis of the MD simulations. Conformation distribution of **(A)** WT and **(B)** A9V/Y13K/Y312R. The active sites adjacent to the MSPS of **(C)** WT and **(D)** A9V/Y13K/Y312R. The protein is represented as a surface in gray. The active sites are represented as sticks. The ligand MSPS is indicated by sphere. Protein interactions with the MSPS of **(E)** WT and **(F)** A9V/Y13K/Y312R. The protein-ligand interactions are categorized into four types: water bridges (blue), ionic (rosy), hydrophobic (purple) and hydrogen bonds (green).

To further explore the catalytic mechanism and interactions between protein and ligand, MD simulations were conducted using the WT and A9V/Y13K/Y312R complexed with MSPS for 50 ns. The root-mean-square deviation (RMSD) showed that the C_α_ and side chains had a relatively small fluctuation range, indicating that the simulation system was stable (Fig. S9). Furthermore, the RMSD of A9V/Y13K/Y312R was lower than that of the WT, suggesting that A9V/Y13K/Y312R exhibited less structural deviation than the WT during the simulation. According to the reported mechanism, the two conserved H89 and H133 residues in l-PpTA might abstract a proton from the substrate hydroxyl group to form a C–C bond and initiate aldol reactions, providing flexibility in recognition of the C_β_ configuration. The NE2_H89_–O_MSPS_ distance in the WT and A9V/Y13K/Y312R complexed with MSPS fluctuated in the range of 2.9–5.4 Å and 2.8–5.1 Å, respectively, while the ND1_H133_–O_MSPS_ distance fluctuated at a much greater length (4.1–7.8 Å and 3.2–5.7 Å, respectively) (Figure 9). These results suggested that H89 was more likely to abstract the proton compared with H133. Furthermore, the ND1_H133_–O_MSPS_ distance in A9V/Y13K/Y312R was much shorter than that in the WT, indicating that the H133 residue in the former had a strong interaction with the MSPS oxygen atom. The interactions among residue side chains and MSPS were further monitored during the MD simulations (Supplementary Figure S10). The A9V/Y13K/Y312R mutant exhibited a reshaped catalytic pocket, changing from a spacious catalytic pocket to a smaller and narrower pocket (Figure 9C; Figure 9D), which resulted in the phenyl and sulfonyl groups of MSPS being firmly anchored in the binding pocket and enhanced more interactions between enzyme and MSPS. Compared with the WT (Figure 9E), residues V9, S10, D11, N12, K13, D93, R177, R312, and R321 showed different degrees of improvement in hydrogen bonding, hydrophobic interactions, and water bridge forces in A9V/Y13K/Y312R (Figure 9F). Furthermore, R312 formed additional hydrogen bonds with the carboxylate groups of MSPS to form a π-cation interaction between the enzyme and benzene ring of MSPS (Supplementary Figure S10B). Therefore, A9V/Y13K/Y312R conferred a higher conversion and C_β_-stereoselectivity toward MSPS compared with the WT.

## Conclusion

To improve the efficiency of the l-PpTA-catalyzed production of MSPS using directed evolution, a simple and fast HTS screening method was constructed by calibrating the aldol reaction activity to detect substrate consumption. The error PCR technique was used to construct the mutant library and a color rendering method with DNPH to screen potential mutations was explored. Five amino acids lining the catalytic pocket were found to improve catalytic efficiency in the aldol reaction direction. Structure-guided SM and ICM were used to increase the catalytic efficiency, and variant A9V/Y13K/Y312R increased the conversion and *de* values by about 2.3-fold and 5.1-fold compared with the WT. Molecular modeling results suggested that the additional hydrogen bonds, hydrophobic interactions, and π-cation interaction between A9V/Y13K/Y312R and MSPS promoted the improved conversion and stereoselectivity. This study provides a promising method for addressing the challenge of engineering TA enzymes to efficiently prepare β-hydroxy-α-amino acids with two chiral sites by monitoring the aldol condensation activity.

## Data Availability

The datasets presented in this study can be found in online repositories. The names of the repository/repositories and accession number(s) can be found in the article/Supplementary Material.

## Abbreviations

DNPH, 2,4-dinitrophenylhydrazine; HTS, high-throughput screening; ICM, iterative combinatorial mutagenesis; MSBA, 4-methylsulfonylbenzaldehyde; MSPS, l-threo-4-methylsulfonylphenylserine; PLP, pyridoxal-5-phosphate; SM, saturation mutation; TA, threonine aldolase.
