# Supplementary material for: Comprehensive screening strategy coupled with structure-guided engineering of l-threonine aldolase from Pseudomonas putida for enhanced catalytic efficiency towards l-threo-4-methylsulfonylphenylserine
Source: Front Bioeng Biotechnol. 2023 Jan 30;11:1117890. doi: 10.3389/fbioe.2023.1117890 (PMC9922994; doi:10.3389/fbioe.2023.1117890)
Supplement: Supplementary file 1 [file DataSheet1.docx]

**Comprehensive screening strategy** **coupled with structure-guided engineering of l-threonine aldolase from *Pseudomonas putida* for enhanced catalytic efficiency towards l-*threo*-4-methylsulfonylphenylserine**

Lihong Li^1^, Rongzhen Zhang^1,^*, Yan Xu^1^, Wenchi Zhang^2^

^1^Lab of Brewing Microbiology and Applied Enzymology, School of Biotechnology and Key Laboratory of Industrial Biotechnology of Ministry of Education, Jiangnan University, Wuxi, 214122, P. R. China

^2^Solomon H. Snyder Department of Neuroscience, Johns Hopkins University School of Medicine, Baltimore, MD 21205, USA

***Corresponding author: Rongzhen Zhang**

**Tel: +86-510-85918201; Fax: +86-510-85864112**

***Email address*: rzzhang@jiangnan.edu.cn**

**Present address: School of Biotechnology, Jiangnan University, 1800 Lihu Avenue, Wuxi City, China, 214122**

Fig. S1 Construction of recombinant plasmids.


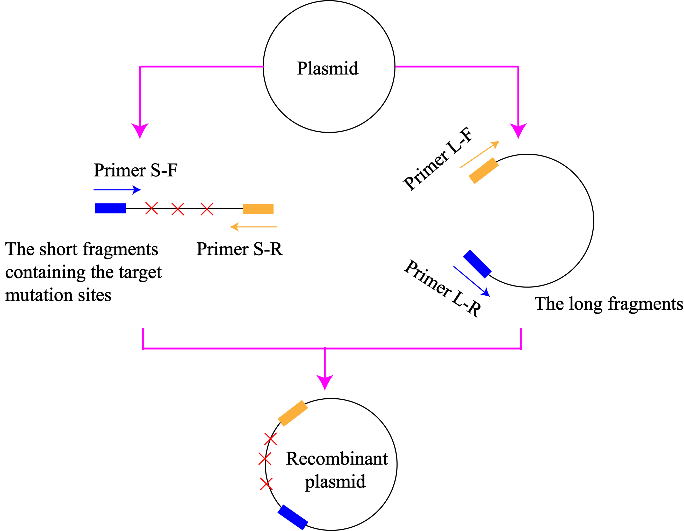


Fig. S2 Workflow of random mutagenesis, screening and characterization of l-PpTA variants using DNPH method.


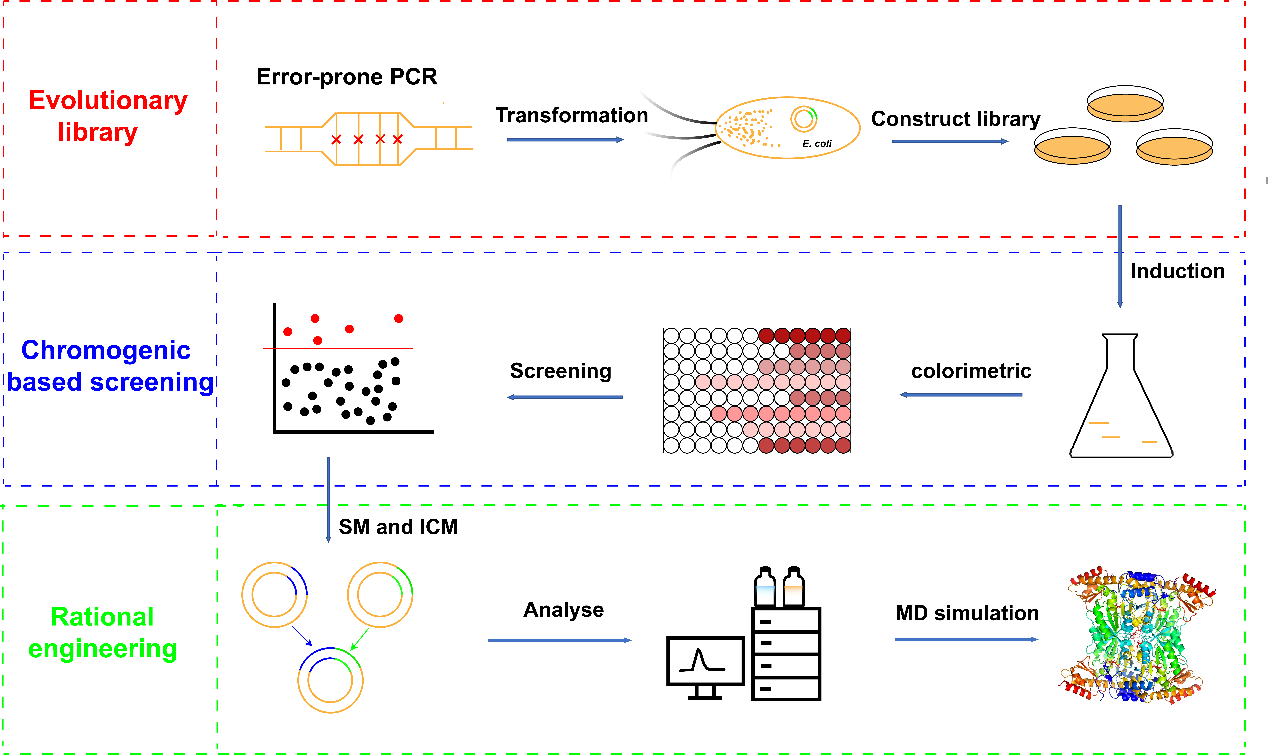


Fig. S3 SDS-PAGE analysis of WT l-PpTA and the variants. Lane M: protein marker; lane 1: supernatant of the WT L-PpTA; lane 2: supernatant of the l-PpTA/A9L; lane 3: supernatant of the l-PpTA/Y13K; lane 4: supernatant of the l-PpTA/H133N; lane 5: supernatant of the l-PpTA/E147D; lane 6: supernatant of the l-PpTA/Y312E;


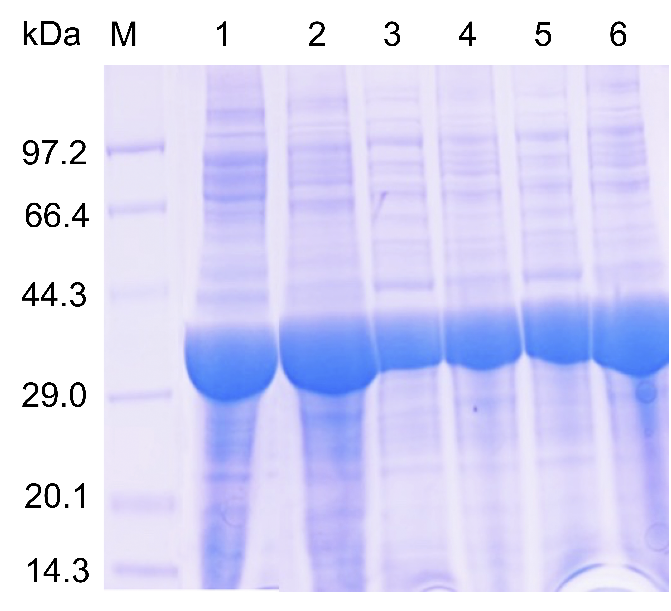


Fig. S4 Screening results of NNK saturation mutagenesis. The beneficial mutants are represented as red dots.


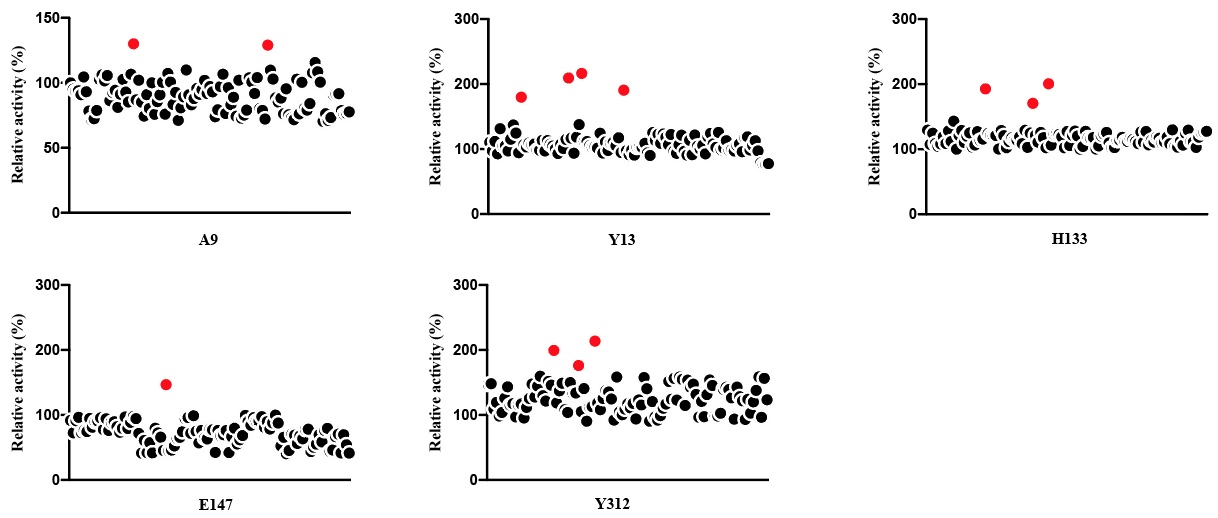


Fig. S5 The conversion and *de* value of WT and its variants of SM and ICM


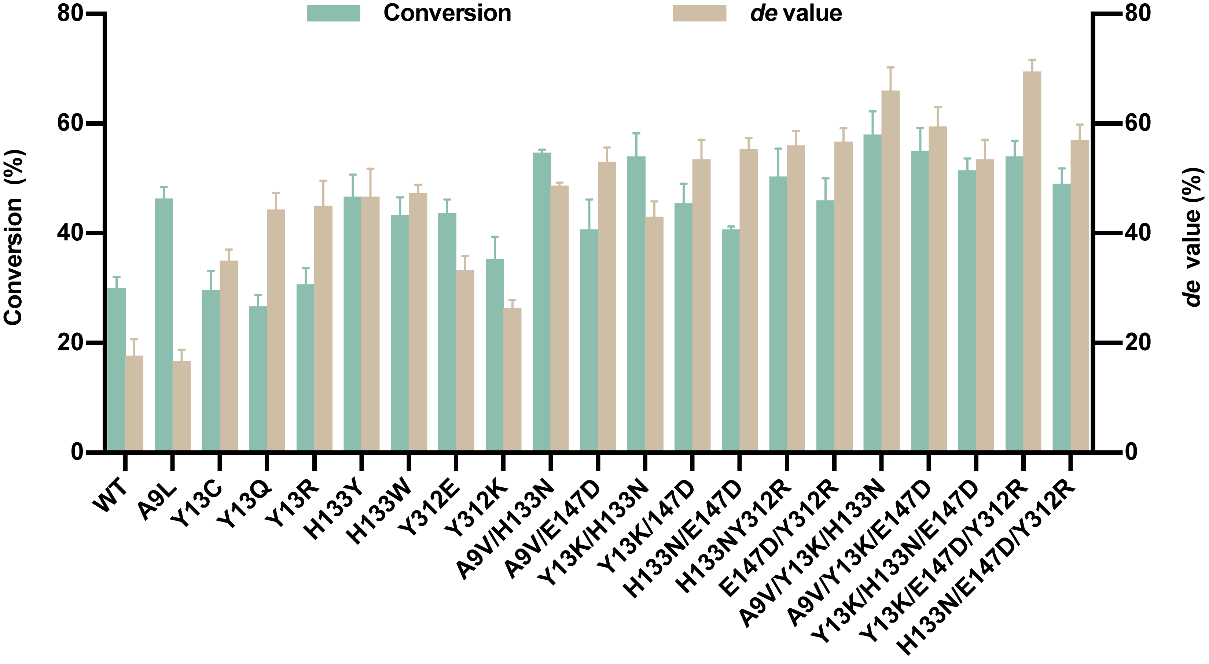


Fig. S6 The HPLC spectra of l-PpTA and the mutant A9V/Y13K/Y312R.


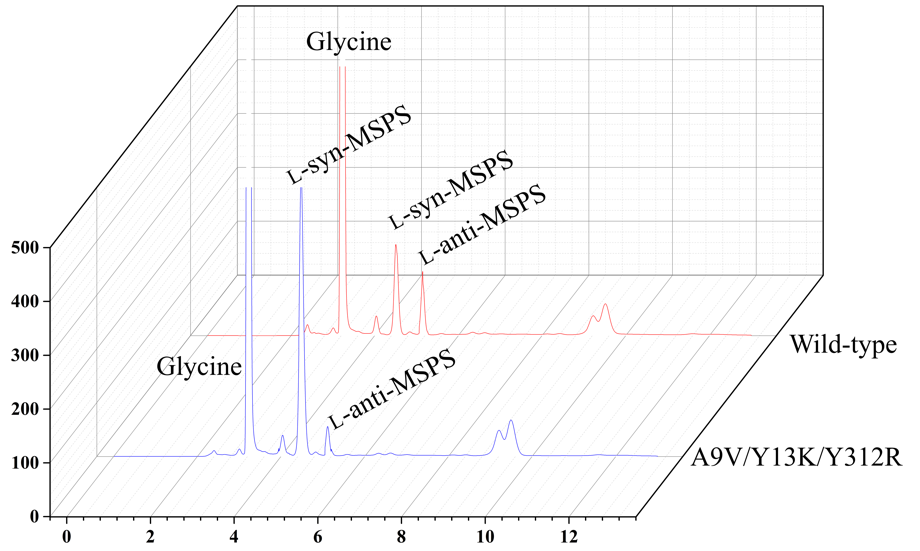


Fig. S7 Sequence alignment of l-TAs from different sources.


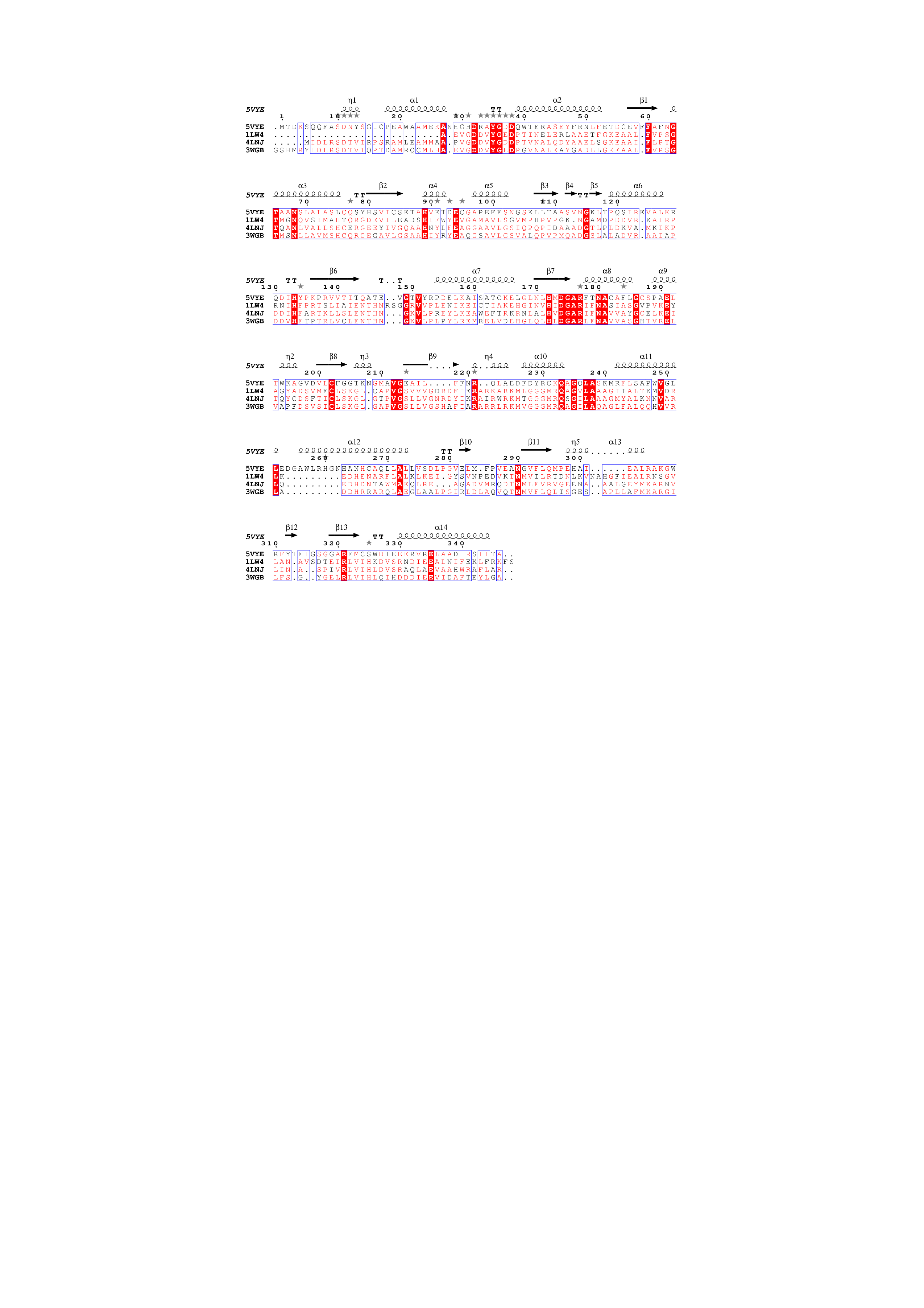


Fig. S8 Flexible docking results of MSPS (cyan) in A9V (A), Y13K (B) and Y312R (C). Active sites and PLP are displayed in grey, the interactions are indicated by red dashes.


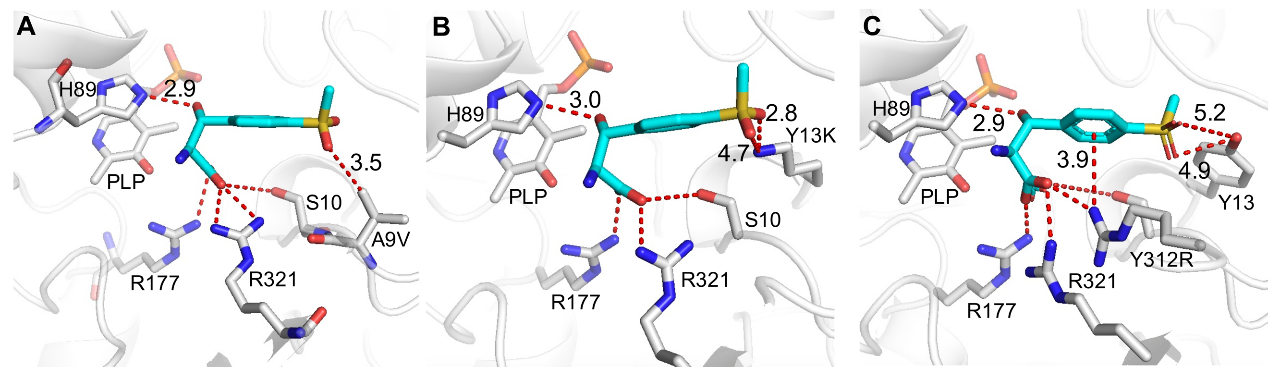


Fig. S9 Molecular dynamic simulations of WT (A) and A9V/Y13K/Y312R (B). The simulation time was 50 ns.


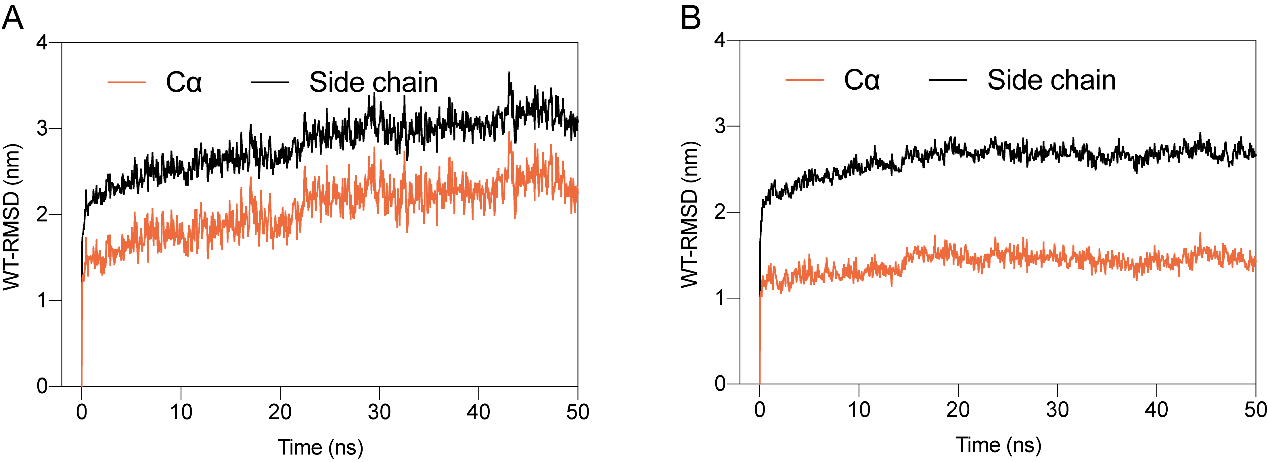


Fig. S10 Interactions analysis of WT (A) and A9V/Y13K/Y312R (B) toward MSPS.


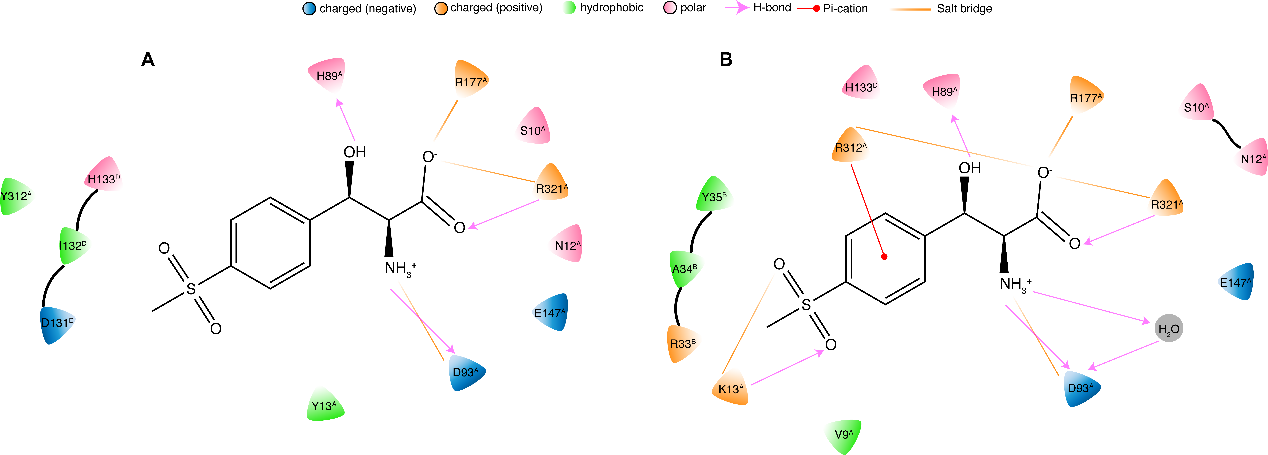


Table S1 Primers used in the site saturation mutagenesis

| Libraries | | Primers (5′-3′) |
| --- | --- | --- |
| A9 | S9-F | ATAAAAGCCAGCAGTTCNNKTCTGATAAC |
|  | S9-R | CTTTGCAGGTCGCAGAGATCGCT |
|  | L9-F | ATCTCTGCGACCTGCAAAGAACTGG |
|  | L9-R | GAACTGCTGGCTTTTATCGGTCA |
| Y13 | S13-F | AGCAGTTCGCGTCTGATAACNNKAGCGGCATC |
|  | S13-R | GCACAGAACATCAACGCCCGCTTT |
|  | L13-F | GGCGTTGATGTTCTGTGCTTCGGCGGTA |
|  | L13-R | TTATCAGACGCGAACTGCTGGCTTTTATC |
| H133 | S133-F | TGAAACGCCAGGATATCNNKTATCCGAAAC |
|  | S133-R | TATAGAAACGCCAACCTTTCCCACG |
|  | L133-F | AAGGTTGGCGTTTCTATACCTTCATCGG |
|  | L133-R | GATATCCTGGCGTTTCAGCGCCACTT |
| E147 | S147-F | TTACCATCACCCAGGCGACCNNKGTTGGCAC |
|  | S147-R | TATCCCAGCTACACATGAAACGAGCACCACC |
|  | L147-F | TCATGTGTAGCTGGGATACCGAAGAG |
|  | L147-R | TCGCCTGGGTGATGGTAACAACACG |
| Y312 | S312-F | AGTGGCGCTGAAACGCCAGGATATCC |
|  | S312-R | CCACCAGAACCGATGAAGGTNNKGAAACGCCA |
|  | L312-F | ACCTTCATCGGTTCTGGTGGTGCTC |
|  | L312-R | TGGCGTTTCAGCGCCACTTCACGGAT |
